# Monthly Reports

**Published:** 1803-08-01

**Authors:** J. Ricards

**Affiliations:** No. 68, Hatton Garden


### MONTHLY REPORTS.

*Cases admitted under the Care of the Surgeon of the Finsbury Dispensary, St. John's Square, Clerkenwell, from June 10, to July 10, 1803.*

| | | | |
|---|---|---|---|
| Ophthalmia - - - - - | 1 | Hydrarthus Genu - - | 2 |
| Phlegmone Testis - - | 1 | Prolapsus Ani - - - - - | 1 |
| Abscessus - - - - - - | 3 | Leucoma - - - - - - | 1 |
| Ulcera Cruris - - - - | 12 | Cataracta - - - - - - | 1 |
| Fistula Lachrymalis - - | 1 | Hematuria - - - - - - | 1 |
| Contusiones - - - - - | 9 | Suppressio Urinæ - - - | 1 |
| Combustura - - - - - | 2 | Spinæ Incurvatio - - | 1 |
| Lues - - - - - - - - - | 3 | Incontinentia Urinæ - | 1 |
| Gonorrhœa - - - - - | 3 | | — |
| Scrophula - - - - - - | 1 | Total - | 45 |

In the practice of Surgery, it is not unfrequent to find more trouble and disagreeable circumstances arising from the treatment of cases which are apparently to a common observer, and especially to the patient himself, of a trivial and unconsequential nature, than from those which are generally considered as cases of the first magnitude. A successful operation for Aneurism, or Lithotomy, where the conduct is plain and void of difficulty, shall often produce a lasting reputation, while an obstinate and insignificant eruption, or some such case, shall frequently baffle all our endeavours, and perhaps bring disgrace upon the practitioner.

From daily observation, it appears to me that the majority of the profession pay too slight a regard to what may be called the Minutiæ of Surgery. Their attention is too much converged to the consideration of those well-marked diseases, which have been regularly classed by Nosologists, while the variety of anomalous appearances which are frequently occurring are not sufficiently noticed. Persons who are unacquainted with the Physiology and Pathology of an animal body, and of the powers of art on the structure and functions of that body, estimate the difficulty of removing a disease as proportionate to the danger connected with it; but it must be well-known to every practitioner, that there are many cases perfectly unconnected with danger which give far greater embarrassment and trouble than diseases of the most formidable aspect.

As there is no disease which is attended with so great a variety of symptoms as syphilis, so there are some of those symptoms symptoms that occasionally happen, which are peculiarly troublesome, and their removal attended with much difficulty; and there are some of those whose existence is not dangerous but rather inconvenient to the patient, and which exist even after the disease (in itself considered) is eradicated. I know of no circumstance more distressing, and productive of more trouble to the surgeon, than that of Warts, which though at first sight they may seem by an unaccustomed observer to be very insignificant, yet are absolutely, in many instances, extremely harrassing; and the more so, inasmuch as a person so afflicted expects their removal to be easy, and is much dissatisfied at their continuance.

I believe that mercury has no influence on these excrescences, and that when all other symptoms are removed, and such a course has been employed as is customary to adopt to eradicate the disease, there is no indication for a further continuance of it on account of warts. It should seem as if there was a peculiar action induced in the parts for the formation of these excrescencies, and when one of these has once arisen, it is astonishing how quickly it increases and how very difficultly it is eradicated, while that disposition remains in the parts; for although it may be acted upon by escharotics, as soon as their action has ceased there is a speedy renewal of the wart. I have seen the most alarming inflammation arise in consequence of the application of powerful caustics, especially when arrived at the root of the wart; and during the existence of that inflammation, the excrescencies have shot forth with redoubled vigour.

In these cases many have much extolled the application of the aq. litharg. acet. undiluted; this often has a very excellent effect in warts of a softer nature, but in many of these, it is altogether without effect.

A case occurred to me lately, where all the usual remedies which are considered as corrosive were used without producing any good effect, and some even seemed to do harm; and these yielded at last to the powder of sabina, which acts by producing a considerable discharge from the surface, by which the excrescence is gradually wasted, without causing an eschar, like a caustic application. This remedy likewise gives little or no pain, and I have never seen it productive of inflammation; which not unfrequently follows the use of either a solution of muriat of mercury or pure potash, or any other of the most potent caustics.

